# Proteorhodopsin variability and distribution in the North Pacific Subtropical Gyre

**DOI:** 10.1038/s41396-018-0074-4

**Published:** 2018-02-23

**Authors:** Daniel K. Olson, Susumu Yoshizawa, Dominique Boeuf, Wataru Iwasaki, Edward F. DeLong

**Affiliations:** 1 Daniel K. Inouye Center for Microbial Oceanography: Research and Education, Department of Oceanography, University of Hawaii, Honolulu, HI 96822 USA; 2 Atmosphere and Ocean Research Institute, The University of Tokyo, Chiba, 277-8564 Japan; 3 Department of Biological Sciences, Graduate School of Science, The University of Tokyo, Tokyo, 113-0032 Japan

## Abstract

Proteorhodopsin is a light-activated retinal-containing proton pump found in many marine bacteria. These photoproteins are globally distributed in the ocean’s photic zone and are capable of generating a proton motive force across the cell membrane. We investigated the phylogenetic diversity, distribution, and abundance of proteorhodopsin encoding genes in free-living bacterioplankton in the North Pacific Subtropical Gyre, leveraging a gene catalog derived from metagenomic samples from the ocean’s surface to 1000 m depth. Proteorhodopsin genes were identified at all depths sampled, but were most abundant at depths shallower than 200 m. The majority of proteorhodopsin gene sequences (60.9%) belonged to members of the SAR11 lineage, with remaining sequences distributed among other diverse taxa. We observed variations in the conserved residues involved in ion pumping and spectral tuning, and biochemically confirmed four different proton pumping proteorhodopsin motifs, including one unique to deep-water SAR11. We also identified a new group of putative proteorhodopsins having unknown function. Our results reveal a broad organismal and unexpected depth distribution for different proteorhodopsin types, as well as substantial within-taxon variability. These data provide a framework for exploring the ecological relevance of proteorhodopsins and their spatiotemporal variation and function in heterotrophic bacteria in the open ocean.

## Introduction

Two principal molecular mechanisms are known to exist in bacteria and archaea for harvesting light energy: chlorophyll-based photosystems and microbial (Type I) rhodopsins [1, 2]. Rhodopsins are retinal-based photoreceptors found in the genomes of bacteria inhabiting both freshwater and marine environments. They are known to be present in members of all three cellular domains of life [3–6] and also viruses [7, 8]. These photoreceptor proteins have evolved to perform several different biological functions including ion transport, light sensing, and gene regulation [9–11]. Proteorhodopsins were the first ion pumping rhodopsins found in bacteria and are now known to be broadly distributed taxonomically and geographically throughout the ocean’s photic zone [12–14]. Most marine bacterial proteorhodopsins characterized so far appear to be proton pumps, are expressed in native marine microbial populations, and are capable of functioning in energy production [5, 15, 16–19]. Therefore, light appears to be commonly utilized as a supplemental energy source in heterotrophic bacterial carbon and energy cycling in the sea.

The current understanding of the light energy flow mediated by proteorhodopsins is limited. The diversity of phylogenetic, genomic, and physiological backgrounds of proteorhodopsins adds to the challenge of understanding the role these molecules play in the lives of various microbes, and emerging data suggest the benefit of proteorhodopsin-based phototrophy occurs through various physiological and ecological strategies. Expression of proteorhodopsins in heterologous hosts such as *Escherichia coli*(*E. coli*) under light and dark conditions have shown light associated increased ATP production [20]. Increased flagellar rotation speeds and increased survival, when grown under respiratory stress, have also been observed [21]. In addition, increased enzymatic activity has been reported when proteorhodopsin is coexpressed with a hydrogenase enzyme [22]. Expression of a SAR11 proteorhodopsin in the Gammaproteobacteria *Shewanella oneidensis* suggests that the proteorhodopsin generated proton motive force leads to increased rates of lactate uptake [23]. Culture experiments with the native proteorhodopsin-containing *Vibrio* sp. AND4 have demonstrated an increase in survival rates under extended periods of starvation when cultures were exposed to light [24]. Subsequent work in various *Vibrio* strains has also shown increased ATP production and increased cell viability under respiratory stress [25]. Studies in the SAR11 affiliated *Candidatus Pelagibacter ubique* showed increased ATP production and ATP-dependent transport of taurine with light under starvation conditions [26]. Although some organisms appear to use proteorhodopsin for enhanced survival under starvation conditions, light-mediated growth stimulation has also been reported for some *Flavobacteria* [27–30]. These studies suggest light-driven proton pumping mediated by proteorhodopsins can enhance various cellular processes directly when dependent upon the proton motive force, as well as providing the energy for ATP production. These data also suggest that different rhodopsin-containing bacteria likely utilize unique light-dependent fitness strategies somewhere along a spectrum between enhanced growth and survival conditions [5].

The seven transmembrane domains in rhodopsin tertiary structure are highly conserved, although amino-acid and nucleotide sequences can possess a great deal of variability [3, 6]. In addition to conserved tertiary structure, several amino-acid residues have been determined important for rhodopsin function. For example, a lysine in transmembrane seven is required for the covalent linking of retinal to the opsin [31]. In addition, variations in the amino acid at position 105 (93 in bacteriorhodopsin numbering) in the third transmembrane domain have been shown to be important for the spectral tuning of the molecule [13, 32, 33]. It has also been observed that a single residue change at amino-acid 105 can alter the rhodopsins efficiency at capturing the light of different wavelengths [33]. Three common variants include blue light absorbing glutamine (Q) and green light absorbing leucine (L) and methionine (M), although additional, less frequent naturally occurring amino-acid substitutions at these sites have also been reported.

In addition to the spectral tuning site in the third transmembrane domain, three residues have been implicated in the ion pumping mechanism of the molecule. The amino-acid motif consisting of residues 97, 101, and 108 (85, 89, 96 with bacteriorhodopsin numbering) are thought to be important in determining the ion pumped by the protein [30, 34–37]. For example, aspartate, threonine, glutamate (DTE) or aspartate, threonine, aspartate (DTD) are associated with proton-pumping rhodopsins [3]. Others such as asparagine, aspartate, glutamine (NDQ) and asparagine, threonine, glutamine (NTQ) are associated with sodium pumps or chloride pumps, respectively [30]. These residues surround the retinal, which is covalently linked to a lysine in transmembrane seven, creating the unique environment for the transport of the various ions by the different types of pumps.

To further characterize rhodopsin distribution and variability in the marine environment, we investigated the phylogenetic diversity, distribution, and abundance of proteorhodopsin groups derived from an extensive catalog of genes assembled from metagenomic depth profiles of free-living bacterioplankton at Station ALOHA in the North Pacific Subtropical Gyre (NPSG) [38]. These data range from the sea surface to 1000 m collected at approximately monthly intervals over one and a half years. Unique proteorhodopsin genes (1510 total) were identified, and the variability of rhodopsin sequence motifs, their expression, and activities were determined. Our new data and analyses highlight unexpected spatial and organismal distributions of different proteorhodopsin types throughout the water column, as well as within-taxon variability of these photoproteins.

## Materials and methods

### **Searching the Station ALOHA gene catalog**

Sequence data originate from the Station ALOHA gene catalog, which was generated as previously reported [38]. Briefly, metagenomic sequencing data from a 0.22 μm bacterial fraction was generated from 83 samples that were prefiltered on 1.6 μm filter to exclude larger organisms and particles. Samples were obtained monthly over a 1.5-year sampling period from August 2010 to December 2011. Seven depths ranging from 25 to 1000 m were sampled at Station ALOHA on 11 cruises of the Hawaiian Ocean Time series. Approximately 40 million protein-coding genes were identified and clustered into 8.9 million non-redundant genes based on 95% nucleotide identity. Sequence data used in this study are available from the NCBI short read archive under Bioproject no. PRJNA352737, and the metagenomic data products (contigs, genes, peptides) are available at iMicrobe (https://imicrobe.us/project/view/263).

In this study, HMMer v 3.0 [39] was used to construct profile hidden Markov models (HMM) using known amino-acid sequences for a gene of interest. Three separate HMM profiles were constructed for proteorhodopsins. The first contained 59 sequences from bacterial genomes obtained from NCBI. The second contained 216 sequences generated using a SAR86 proteorhodopsin to search the EMBL HMMER database [40]. The third was generated by downloading ~7900 manually curated sequences of type-1 rhodopsins from MICrhoDE database [41]. All three HMMs were used to search the Station ALOHA gene catalog [38] and results compared. Significant hits were then annotated using eggNOG [42]. Putative proteorhodopsin sequences were further assessed by sequence alignments and phylogenetic analysis with a set of 120 reference sequences (Supplemental File 1). Potential novel rhodopsin sequences were analyzed to identify the seven transmembrane domains and retinal binding lysine, which define opsin genes. Sequences lacking seven transmembrane domains or the retinal binding lysine in the seventh transmembrane domain were excluded prior to downstream analysis of the data set. All sequences used in the subsequent analysis (Supplemental File 2) had a top eggNOG annotation of rhodopsin. Searches for retinal biosynthesis genes were conducted in a similar manner as with the rhodopsin genes using similarly prepared HMM profiles.

### Abundance estimations

The abundance of genes relative to total genome equivalents were calculated in Mende et al. [38]. Briefly, the average gene copy per genome was calculated by taking all coverages and dividing by the average coverage of 10 universal single-copy genes (COG0012, COG0016, COG0018, COG0172, COG0215, COG0495, COG0525, COG0533, COG0541, and COG0552) found in the samples. Metagenomic operational taxonomic units (mOTUs) are defined as species-level sequence clusters of a set of near-universal, single-copy protein-coding genes recovered from metagenomic datasets [43, 44]. A customized version of the mOTUs was constructed in Mende et al. [38]. Statistical analysis included analysis of variance and *t*-tests and was performed in R.

### Sequence alignment and phylogenetic analysis

All sequence alignments were done with Clustal Omega [45] using default settings. Alignments were visualized and inspected with Jalview [46]. Phylogenetic analysis was performed with the ETE2 toolkit [47] utilizing the clustalO_trimal_raxml:bootstrap workflow. In all, 120 reference sequences (Supplemental File 1) were used as controls in the tree. Tree visualization was performed in iTOL [48].

### Taxonomic annotation

The Station ALOHA gene catalog was annotated as previously described [38]. Briefly, genes were assigned taxonomic annotations utilizing LAST version 756 with scoring parameters “-b 1 -x 15 -y 7 -z 25” to align sequences to an augmented version of RefSeq release 75 that had been amended by a number of high-quality SAGs from marine environments. Each gene was assigned to the most specific taxon common to all RefSeq hits scoring within 1% of the best hit. Genes were assigned to the KEGG orthologous group or groups represented by all KEGG genes scoring within 5% of the best hit. The HMM-based eggnog-mapper tool was used to obtain eggNOG annotations. Here, gene catalog annotations were compared with the clustering of sequences with reference sequences in the phylogenetic tree. Additional analysis was done for sequences in which phylogenetic analysis disagreed with the gene catalog annotations such as those from giant viruses that lack reliable annotations in RefSeq. This additional analysis was done using protein-protein BLAST to search non-redundant protein sequences with rhodopsin sequences of interest, as well as neighboring genes found on the contigs to confirm taxonomic affiliation.

### Motif analysis

The 1510 representative rhodopsin sequences in the Station ALOHA gene catalog represent 6682 total sequences found in the original metagenomes that were clustered based on 95% nucleotide identity. For analysis of single amino-acid variation, all 6682 sequences were used. All Station ALOHA proteorhodopsins were aligned with reference rhodopsins and screened for the presence of amino acids at positions 97, 101, 105, and 108. Partial sequences lacking these sites were not included in the motif analysis.

### **Proteorhodopsin gene expression**

RNA extractions, library preparation, sequencing, and bioinformatic analysis were performed as previously described [49]. Briefly, 2 liters of seawater for a given sample were filtered through 25 mm 0.2 μm Supor PES Membrane Disc filters (Pall, USA) housed in Swinnex units and filters placed in RNALater (Ambion, Grand Island, NY) immediately afterward and preserved at –80°C until processing. RNA extractions were performed by removing RNALater followed by the addition of 300 μl of Ambion denaturing solution directly to the filter and then vortexed for 1 min. Prior to purification, 750 μl of nuclease-free water was added. Samples were purified, and DNase treated using Chemagen MSM I instrument with the tissue RNA CMG-1212A kit (Perkin Elmer, Waltham, MA). RNA quality was assessed using the Fragment Analyzer high sensitivity reagents (Advanced Analytical Technologies, Inc.) and quantified using Ribogreen (Invitrogen, Waltham MA). Metatranscriptomic libraries were prepared for sequencing with the addition of 5–50 ng of total RNA to the ScriptSeq cDNA V2 library preparation kit (Epicentre, Chicago, IL).

Metatranscriptomic samples were sequenced with an Illumina Nextseq500 system using V2 high output 300 cycle reagent kit with PHIX control added for metatranscriptomic (5%) libraries [50]. Sequencing reads were trimmed using Trimmomatic v. 0.27 (parameters: ILLUMINACLIP::2:40:15 [51]), end-joined using PandaSeq v. 2.4 (parameters: -F -6 -t 0.32, quality cutoff of 0.32 [52]), and quality-filtered using sickle v. 1.33 (length threshold set to 50 [53]). Reads mapping to ribosomal RNA (rRNA) were then removed using sortmerna v. 2.140 to arrive at the final set of joined non-rRNA reads. These reads were mapped to the non-redundant set of genes in the Station ALOHA gene catalog [38] using LAST, and a 95% ID cutoff was used to ensure high-quality mapping.

Deep-water (200–1000 m) proteorhodopsin gene transcripts were acquired from the same water samples in which the metagenomes originated [38]. Near surface water (15 m) proteorhodopsin gene transcripts originated from Wilson et al. [49] as we did not have expression data for surface water samples associated with the Station ALOHA gene catalog above 200 m [38].

### Heterologous expression and functional characterization of representative rhodopsins

All rhodopsin genes used  in heterologous expression studies were codon optimized and chemically synthesized by Eurofins Genomics (Tokyo, Japan, Supplemental Table 1 and 2). These fragment DNAs were inserted into *Nde*I and *Xho*I sites of pET21a vector (Novagen, Madison, WI, USA) to express the rhodopsin in *E. coli* C41(DE3, Lucigen). All rhodopsins were overexpressed in cells grown at 37 °C in 200 ml of 2xYT medium and induced at an optical density (600 nm) of 0.4–0.6 with 0.1 mM IPTG and 10 μM all-trans-retinal. The rhodopsin-expressed cell suspension then was placed in darkness and illuminated using a 300 W xenon lamp (MAX-303; Asahi Spectra, Tokyo, Japan) for 3 min. Light-induced pH changes were measured using a pH meter (LAQUA F-72; Horiba, Kyoto, Japan). The measurements were then repeated under the same conditions after the addition of the protonophore carbonyl cyanide 3-chlorophenylhydrazone (CCCP, final concentration, 10–30 μM). All measurements were performed in 100 mM NaCl at 4 °C.

### Principal component analysis

Eighty-three samples were used as observations to construct a Pearson’s correlation matrix of rhodopsin abundance, biotic parameters (GC content, C-ARSC, N-ARSC, and average gene length) and ancillary data (depth, temperature, salinity, oxygen concentration, dissolved inorganic carbon, pH, alkalinity, and phosphate, nitrates and silicates concentrations). The analysis of principal components was performed using XLStat version 2009.1.02 (Addinsoft).

## Results

### Abundance and distribution of proteorhodopsins at Station ALOHA

To survey proteorhodopsins in the environment, we leveraged a recently constructed gene catalog from a time series metagenomic survey of bacterioplankton from 25 to 1000 m in the NPSG at Station ALOHA [38]. The original sampling was performed at a total of seven depths (25, 75, 125, 200, 500, 770, and 1000 m) approximately once a month for one and a half years [38]. Hidden Markov models were developed to identify all rhodopsin-like sequences in the catalog and assess the abundance, taxonomic affiliations, and distribution of these photoproteins across space and time.

**Fig. 1:**
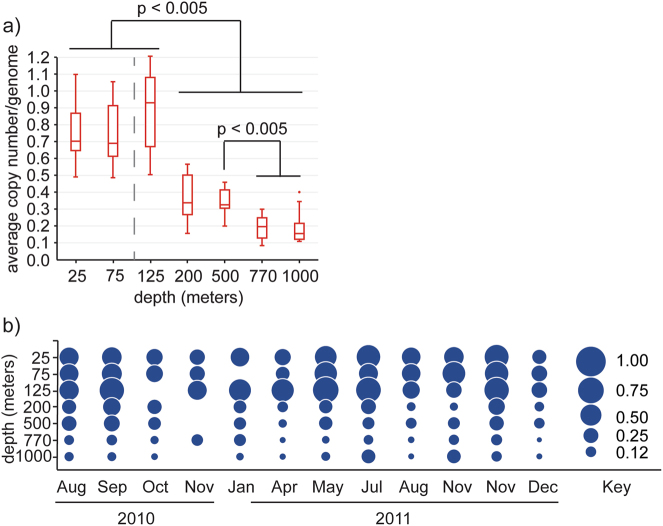
Proteorhodopsin genes were most abundant in near surface waters but present as deep as 1000 m. **a** Box and whiskers plot of average copy number per genome of all proteorhodopsins found at each depth in the Station ALOHA gene catalog. The average deep chlorophyll maximum of 106 m from this sampling period is depicted as a dashed line. Statistically significant differences in abundance between samples are shown. **b** Bubble plot showing average copy number per genome of proteorhodopsins in all samples of the station ALOHA gene catalog. The absence of a bubble represents a lack of sample. Sampling occurred twice in November 2011. 3 November and 27 November

A minimum estimate of 1510 unique proteorhodopsin genes were identified in a catalog of approximately 8.9 million non-redundant gene sequences (95% nucleotide ID) originating from Station ALOHA. Regarding amino-acid identity, there were 1490 unique sequences at 100% amino-acid identity, and 862 unique protein sequences at 95% amino-acid identity. Proteorhodopsins were identified in all samples and at all depths (Fig. 1). Not surprisingly, the majority of proteorhodopsins occurred between depths of 25 and 125 m. At these depths, the proteorhodopsin genes had an average copy number in the community of close to 0.7 per genome at 25 and 75 m, with a slight increase of approximately 0.9 per genome at 125 m. A sharp decline in abundance per genome equivalent was observed at and below 200 m. The average copy number per genome was about 0.34 at 200 and 500 m. A further decrease in abundance was observed at the greater depths of 770 and 1000 m where proteorhodopsins occurred at approximately 0.2 and 0.15 copies per genome, respectively (Fig. 1a).

The general trend of greatest abundance at 25–125 m followed by a decline at 200–1000 m was consistent throughout the sampling period although significant variation was evident in the total amount of proteorhodopsins present in a given month, as well as the relative abundance across the depths for each month (Figs. 1a, b). The proteorhodopsins found at greater depths were unique and depth specific and did not correspond to those found in surface waters (see below). Principal component analysis was performed to identify any physicochemical or biological drivers of possible seasonal variation in rhodopsin abundance (Supplemental Fig. 1). No seasonal correlations were identified.

### Rhodopsin sequence diversity and taxonomic affiliation

**Fig. 2:**
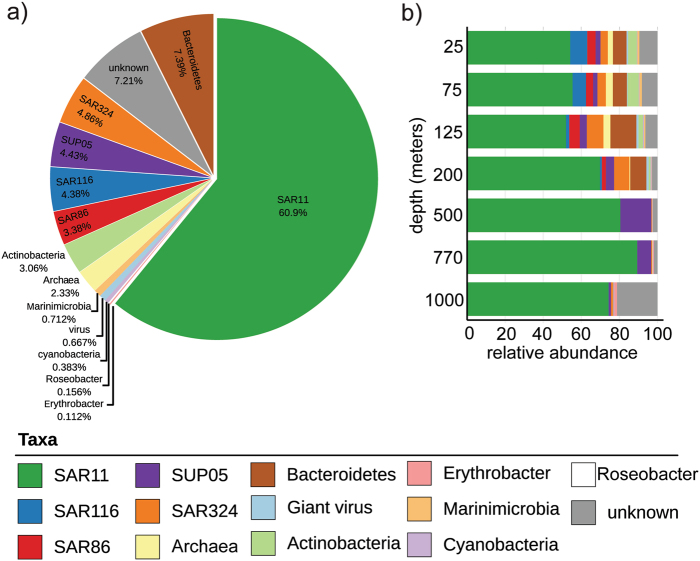
The majority of proteorhodopsin sequences originated from SAR11. **a** Pie chart depicting percent abundance of proteorhodopsin sequences associated with a given taxa. **b** Stacked bar chart representing the relative abundance of proteorhodopsin sequences associated with a given taxa at each depth

The majority of proteorhodopsin sequences found in the bacterial fraction at Station ALOHA (60.9%) appeared to originate from SAR11 clade bacteria, one of the most abundant bacterial groups found throughout the water column (Fig. 2). Approximately 7% of the sequences appeared to originate from Bacteroidetes, whereas another 7% could not be confidently associated with any specific taxa. Additional rhodopsin sequences (~2–5% of the total from each of the following subgroups) were affiliated with rhodopsins found in SAR324, SUP05, SAR116, and SAR86 bacterial clades, Actinobacteria and Euryarchaea. Marinimicrobia, giant virus types, and cyanobacteria, Roseobacter and Erythrobacter-associated sequences accounted for <1% each of the total rhodopsin sequences recovered (Fig. 2a).

SAR11-associated rhodopsin sequences were the most abundant sequences recovered at every depth. The majority of sequences associated with taxa other than SAR11 were primarily found at 200 m and shallower, except for a small percentage from SUP05 and Erythrobacter-associated sequences, which were found at greater depths (Fig. 2b).

### Spectral tuning

**Fig. 3:**
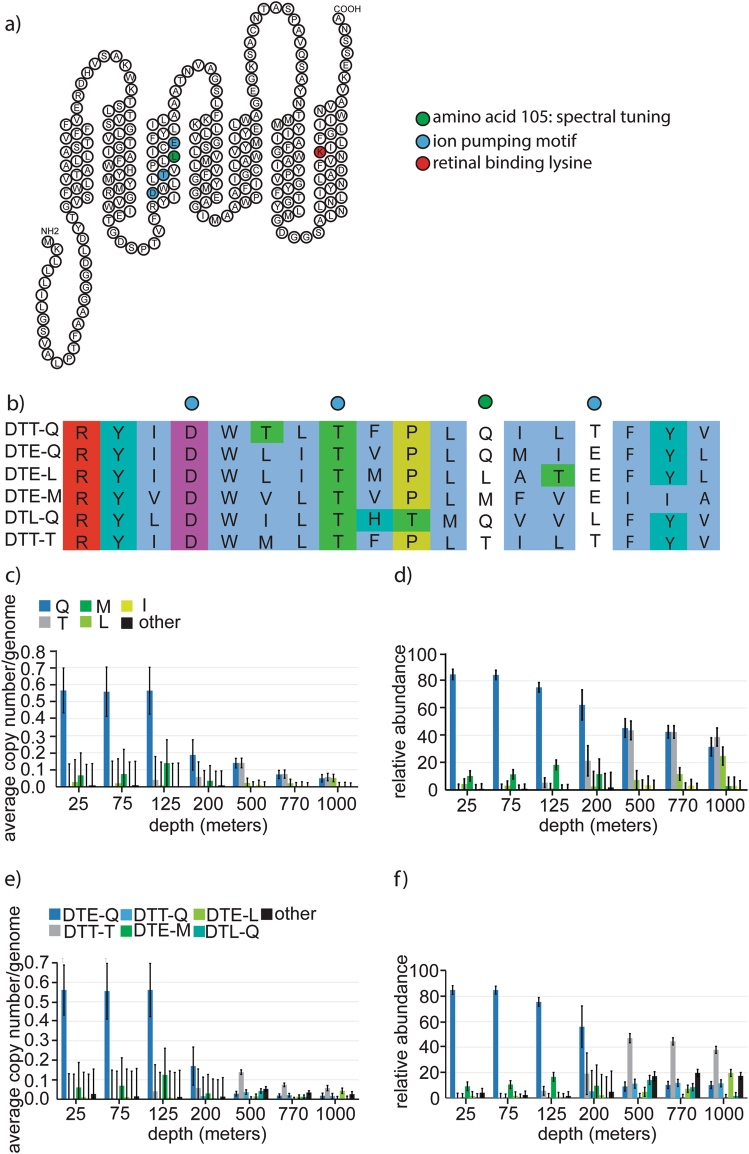
Diversity and abundance of proteorhodopsin sequence motifs. **a** Topology of a representative proteorhodopsin highlighting key residues in retinal binding, spectral tuning, and ion pumping. **b** Sequence alignment of transmembrane peptide segment three, comparing six proteorhodopsin variants highlighting variation in residues involved in spectral tuning and ion pumping. **c** Average copy number of rhodopsins per genome in the metagenome. **d** Relative abundance of spectral tuning variations in proteorhodopsins. **e** Average copy number per genome. **f** Relative abundance of variants in ion pumping motif and spectral tuning site in proteorhodopsins. **c**–**f** Blue, green, and gray color codes represent blue light absorbing, green light absorbing or unknown spectral absorption based on the spectral tuning residue of known proteorhodopsin variants. DTE is known to be a proton-pumping variant

Specific amino-acid residues are known to be critical to rhodopsins (Fig. 3a) with respect to their spectral absorbance and ion transport functional capabilities. To capture all of the variation in single amino-acid variations, we analyzed not just the 1510 unique representative sequences, but all variant rhodopsin genes sequences within the 1510 clusters. Of these 6682 rhodopsin sequences, 4483 included amino-acid 105, which has been implicated as being critical in spectral tuning [33]. We identified 14 amino-acid variants at this site, although 9 of the variants were rare and were only identified in 1–13 sequences (Supplemental Table 3). The following five most abundant variants of amino-acid 105 were identified: glutamine (Q, 2440 sequences), threonine (T, 1440 sequences), leucine (L, 252 sequences), methionine (M, 200 sequences), and isoleucine (I, 109 sequences, Supplemental Table 3). It has been suggested that L, M, and I residues at position 105 absorb maximally in the green light spectrum, whereas Q is associated with blue light absorption [33]. Although T at position 105 has previously been reported in environmental samples, its function with respect to spectral absorption is currently unknown.

We analyzed the normalized abundance and distribution of proteorhodopsins containing different variations of residue 105. At depths between 25 and 125 m, blue light absorbing Q_105_ was most abundant, accounting for ~80% of all proteorhodopsins at these depths. Green absorbing M_105_ was second most abundant at depths 25–125 m, and accounted for about 5–20% of the total proteorhodopsin sequences in these samples, with a maximum average copy number per genome of about 0.1 at 125 m. Green absorbing rhodopsins appear much less abundant than blue absorbing Q_105_ sequences (Figs. 3b–d).

Interestingly, as the abundance declines drastically at 200 m, the relative abundance of Q_105_ (and M_105_) decreased as another amino-acid, T_105_, increased in abundance. At 500–1000 m, the T at the residue in position 105 accounted for approximately 50% of proteorhodopsin sequences at these depths (Figs. 3b–d).

### Ion pumping residues

In addition to amino-acid residue 105, three additional residues (97, 101, and 108) have been implicated in the ion pumping activity of proteorhodopsins (Fig. 3a). We identified 4292 sequences containing 35 different variations of these three residues. Of these, 4256 complete sequences containing both the ion pumping and spectral tuning sites yielded a total of 33 variations of frequency tuning and ion pumping residues identified in the data set (Supplemental Table 3).

The two most abundant variations of the ion pumping motifs were DTE (as previously defined) and aspartate, threonine, threonine (DTT) (Supplemental Table 3 and Figs. 3b, e, f). DTE is believed to be associated with proton pumping while the function of DTT is so far unknown. DTE was primarily found at depths between 25 and 200 m and is associated with Q and M at residue 105. A lesser amount of DTE containing sequences were found in deeper waters. The DTT motif, on the other hand, was found predominantly at depths of 200–1000 m and was associated primarily with T_105_, although it was also found with additional residues such as Q_105_ and M_105_ at a much lesser frequency.

### Phylogenetic analysis

**Fig. 4:**
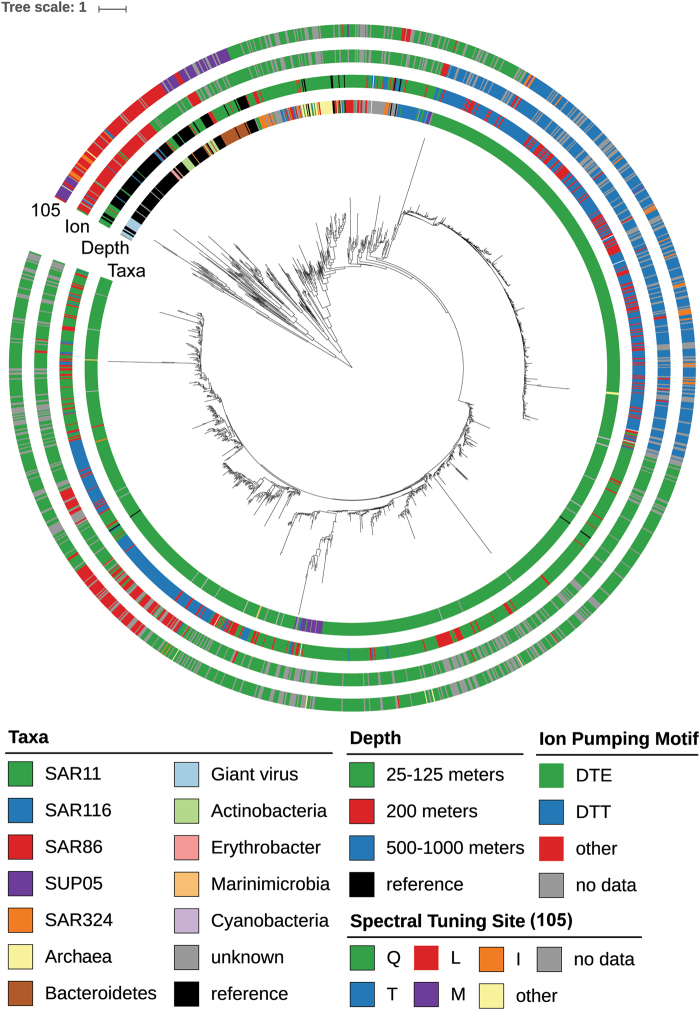
Phylogenetic tree o`f all 1510 unique proteorhodopsin gene sequences plus reference sequences. Associated taxa, depth, ion pumping motif, and spectral tuning site are color coded for each sequence

Phylogenetic analyses were performed utilizing the ETE2 toolkit [47] to determine the relationships between the different rhodopsin gene sequences. A total of 1510 representative rhodopsin gene sequences were aligned along with 120 reference rhodopsins of known phylogenetic origin. Three major branches can be seen, with the majority of the rhodopsin sequences associated with taxa other than SAR11 clustering together on one major branch. The two additional highly populated branches contain mostly SAR11 clade-associated rhodopsins (Fig. 4). The majority of rhodopsins were blue light absorbing proton pumps from depths of 25–200 m affiliated with SAR11 rhodopsins. Additional sequences from the taxa other than SAR11 also appeared to be primarily blue light absorbing proton pumps, with a few green light absorbing proton pumps also represented. Of interest in the near surface waters were rhodopsins most closely related to giant virus rhodopsins, which contained methionine or leucine at position 105 suggesting green light absorption, and novel residues in the ion pumping motif (DTS_L, DTS_M, DTV_M, and DTT_M). Rhodopsin gene sequences originating from 200 to 1000 m, appeared primarily derived from SAR11, and mostly containing the functionally uncharacterized DTT-T motif. A few blue light absorbing proton pumps and some rhodopsin sequences containing unique ion pumping and spectral tuning residues were also found at greater depths.

In addition to SAR11-associated rhodopsin sequences in the deeper waters, there were also a few sequences from SUP05 that were either blue light absorbing proton pumps or unique/novel variants, as well as sodium and chloride pumping *Erythrobacter*-related sequences, which accounted for about 0.1% of the total sequences (Fig. 4).

### Functional characterization of proteorhodopsin genes

**Fig. 5:**
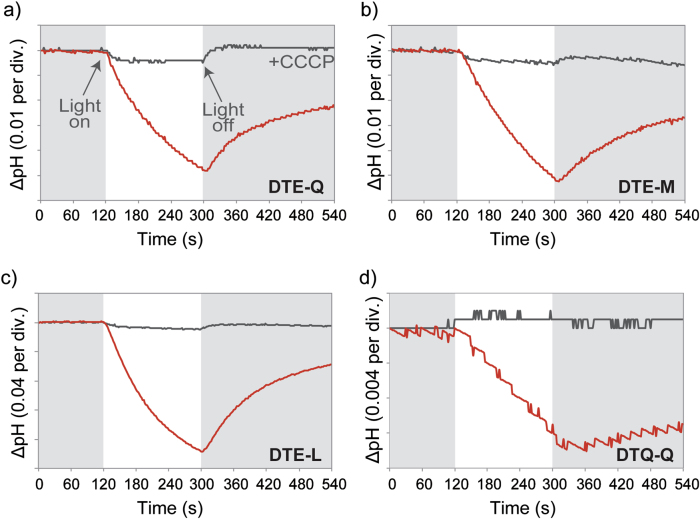
Heterologous expression and proton-pumping activity of representative proteorhodopsin genes recovered in this study. Light-induced pH changes of *E. coli* cell suspensions expressing **a)** DTE-Q, **b)** DTE-M, **c)** DTE-L, or **d)** DTQ-Q rhodopsins in 100 mM NaCl (red line). The pH changes after addition of the protonophore CCCP are indicated (black line)

We tested ion pumping functionality in 20 unique Station ALOHA rhodopsin sequences by expressing synthesized genes in *E. coli* in the presence of retinal and observing pH changes upon illumination (Fig. 5 and Supplemental Table 1).

The majority of the shallow water-associated sequences contained motifs of known function. SAR11 DTE-Q and SAR324 DTE-L motif containing sequences compared with Bacteroidetes DTE-M sequences are believed to be blue light and green light absorbing proton pumps respectively. Consistent with this, we observed a decrease in pH upon illumination due to active pumping of protons across the bacterial membrane (Figs. 5a–c).

The SAR11 DTT-T rhodopsins were the most abundant types found at depths below 200 m, but their function is currently unknown. These rhodopsins, when expressed in *E.coli*, did not elicit a change in pH upon illumination, nor did they appear to bind retinal based on pigmentation in bacterial pellets (Supplemental Tables 1 and 2 and Supplemental Figs. 2 and 4). The lack of retinal binding may be due to a lack of photoprotein functionality, or possibly a lack of proper folding or membrane insertion in *E. coli* recombinants. Interestingly, these deep SAR11-derived rhodopsin sequences lacked a portion of the protein N-terminus corresponding to the signal peptide region, in comparison with surface water associated DTE-Q containing homologs (Supplemental Fig. 3). The addition of the N-terminus from DTE-Q containing shallow water proteorhodopsin to these sequences in synthetic gene constructs did not impart functionality to the DTT-T rhodopsin when expressed in *E. coli* (Supplemental Fig. 4). Also, changing two residues in a functional DTE-Q or DTE-L sequence to DTT-T, resulted in the loss-of-function for these rhodopsins when expressed in *E. coli*, suggesting the DTT-T sequences do not function as proton pumps in surface water rhodopsin sequences under these assay conditions.

Although DTT-T sequences were the most abundant of the deep-water rhodopsin sequences, many other deep-water rhodopsin sequence variants were observed. Most rhodopsin sequences tested from deep-water samples did not exhibit a change in pH and did not appear to bind retinal (Supplemental Table 1 and Supplemental Fig. 2) in vitro. One exception was the SAR11-associated DTQ-Q sequence from 770 m, which when heterologously expressed in *E. coli* exhibited the expected decrease in pH upon illumination (Fig. 5d).

### **Proteorhodopsin gene expression in the mesopelagic**

An important consideration regarding the abundance profiles of proteorhodopsins presented here is that these data only reflect genome and gene abundances and lack information about whether these genes are expressed in situ. It has been previously shown that proteorhodopsins can be highly expressed in surface waters [19, 54], but the level of proteorhodopsin expression for greater depths is less well documented.

**Fig. 6:**
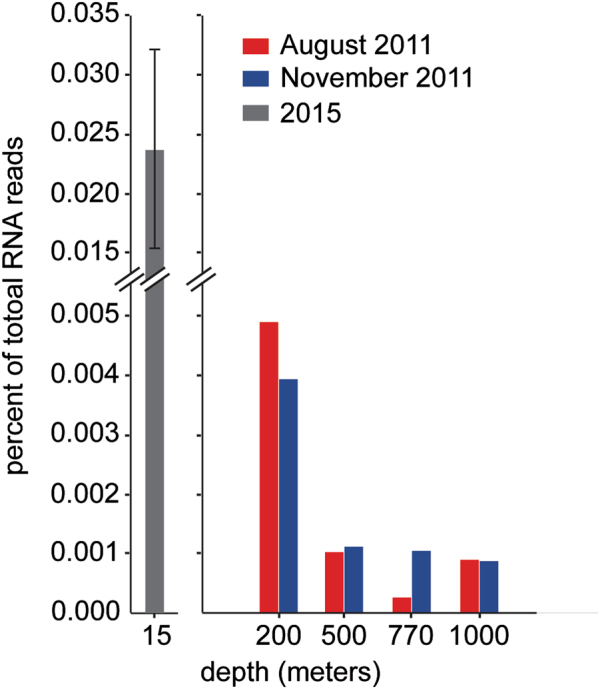
Putative proteorhodopsins were expressed in the deep-water samples. Rhodopsin transcripts normalized to total RNA reads from depths of 200–1000 m in August 2011 (red) and November 2011 (blue). For comparison, the average percentage of total RNA reads for proteorhodopsins in near surface waters (15 m) from a diel sampling scheme in 2015 are depicted in gray [49]

To further explore rhodopsin expression at greater depths, RNA from deep-water samples from depths of 200, 500, 770, and 1000 m in August and early November of 2011 were determined. Although rhodopsin transcripts were detected in all samples, the relative number of rhodopsin transcripts detected was very low at depths of 500–1000 m (Fig. 6), compared with the relative transcript abundances observed at 200 m, as well as samples collected at 15 m from July to August 2015 [49]. Proteorhodopsin transcripts in near surface water samples from Wilson et al. [49] averaged approximately 0.02% of total microbial community transcript abundance, which was comparable to previous reports [19, 54]. A much lower rhodopsin transcript abundance was observed in deeper water samples, ranging from 0.004% at 200 m down to approximately 0.001% of total transcripts at 500–1000 m. This decrease in relative abundance of transcripts by approximately 95% from near surface waters to 1000 m was not unexpected because the total proteorhodopsin gene abundance decreased by approximately 75% at these depths. Nevertheless, significant rhodopsin transcript abundance was observed at all depths.

Analysis of taxon affiliation revealed the majority of these natively expressed proteorhodopsins were affiliated with the SAR11 lineage (Supplemental Fig. 5a). Many of the rhodopsin transcripts mapped to sequences of unknown origin, especially at 500–1000 m (Supplemental Fig. 5a). Analysis of the ion pumping and spectral tuning residues revealed that many contained the blue light absorbing, proton-pumping DTE-Q, as well as the green light absorbing DTE-L and DTE-M motifs (Supplemental Fig. 5b). Interestingly, unique sequences, such as DTT-T, DTT-Q, DTQ-Q, and DTQ-L, were also expressed, especially in the bacterioplankton communities found at 500–1000 m (Supplemental Fig. 5b). Although expression levels of proteorhodopsin in the deep-water metatranscriptomes were lower than at the surface, they were readily detectable supporting a possible functional role for these proteins in deep waters.

### **Distribution of retinal biosynthetic genes**

**Fig. 7:**
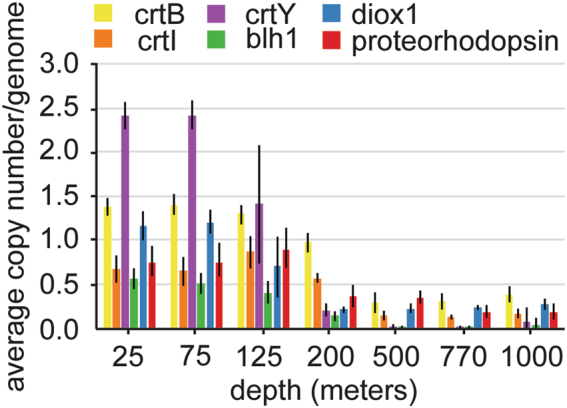
Distribution of retinal biosynthesis genes. Average copy number per genome of crt genes involved in retinal biosynthesis pathway, diox1, and proteorhodopsin at each depth sampled

Proteorhodopsins require retinal to be functional. Retinal is typically produced as an end product in the carotenoid biosynthetic pathway via the cleavage of β-carotene by 15,15’ -β-carotene dioxygenase (*blh* [55]). The* blh* gene homologs in our samples were reasonably abundant in shallow waters (25–125 m), roughly matching observed rhodopsin abundance patterns, but they declined significantly at depths of 200–1000 m (Fig. 7). This suggests a lack of in situ retinal biosyntheses in deeper waters. It is possible these pigments are provided at depth by sinking particles from the surface, or alternative pathways of retinal biosynthesis that require precursors such as phytoene, lycopene, or β-carotene. Significant phytoene and lycopene production might occur at depth because the enzymes responsible for their biosynthesis (crtB and crtI) were found in both shallow and deep-water samples. In contrast, β-carotene and retinal biosynthesis genes crtY and blh are primarily in shallower waters (Fig. 7).

Alternative pathways for retinal biosynthesis have been proposed by Ruch at al. [56] in which apo-carotenoids are converted to retinal by the carotenoid oxygenase Diox1. We investigated the abundance and distribution of putative Diox1 genes in our samples and observed trends similar to that of proteorhodopsin, especially in the deeper samples (Fig. 7).

## Discussion

The discovery of proton-pumping proteorhodopsins in marine planktonic bacteria has expanded our view of the role of retinylidene photoproteins in marine ecosystems. A variety of studies have reported the phylogenetic range, distributions, and sequence diversity of these photoproteins in different environmental contexts. Here, we exploited a large catalog of genes derived from the assembly of metagenomic data sets in depth profiles of free-living bacterioplankton at Station ALOHA [38], to more deeply explore the phylogenetic diversity, distribution, and abundance of proteorhodopsins at greater temporal and depth resolutions.

The majority of 1510 proteorhodopsin sequences identified in our data set occurred between 25 and 125 m depth. The abundance of these proteorhodopsin genes varied month to month relative to the total community gene pool. However, we did not uncover any significant correlation of changes in rhodopsin gene abundances with seasonal variation. Compared with other oceanic regions, Station ALOHA exhibits relatively low seasonality [57, 58]. The stratified surface waters create oligotrophic conditions year-round. Correlations between alpha diversity and seasonally driven environmental parameters such as temperature and mixed layer depth have not been observed at Station ALOHA. Alpha diversity metrics have been reported to consistently correlate with wind speed at 25 m depth 3–10 days prior to sampling, and beta diversity has been shown to correlate with average solar irradiance [58]. Consistent with previous reports, we did not observe major shifts in physiochemical properties at Station ALOHA during the sampling period [38].

Of these 1510 proteorhodopsins, most were blue light absorbing proton pumps with >50% originating from SAR11 clade bacteria. Both blue and green light absorbing proton-pumping type rhodopsins from numerous additional taxa were also identified. In addition to bacterial taxa, we found proteorhodopsins associated with giant viruses [7, 8].

At Station ALOHA, both GC content and genome size have been found to increase at and below the base of the photic zone, with the smaller genomes occurring in surface waters of the mixed layer [38]. This sharp genomic transition zone (GTZ) appears to be driven by nutrient limitation as the small genome size and low GC content at the surface corresponds to lower nitrogen content in the genome and the predicted proteome. Peaks in microbial community diversity were also reported to occur through the GTZ, at the 75–125 m, 125–200 m, and 200–500 m depth horizons. With regard to community richness, the lowest number of different mOTUs were found close to the surface, with a peak at 125 and 200 m, and a subsequent drop with increasing depth [38]. Below 200 m, SAR11 and SUP05 were dominant among PR-containing bacteria, consistent with the distributions of these taxa in the water column. The increased diversity and abundance of proteorhodopsins between 0 and 200 m corresponded with the high-light levels found near the surface and was also consistent with the greater bacterioplankton taxon diversity that occurs at these depths [38].

Although the majority of proteorhodopsin genes were found above 200 m, putative proteorhodopsin sequences were identified as deep as 1000 m, and transcriptomic analyses confirmed these genes were expressed at depth. Sunlight penetrates very deep into the open ocean and becomes approximately equal to endogenous background bioluminescence at around 600 m at Station Aloha [57]. It is possible that proteorhodopsins could operate even at these lower photon fluxes, and contribute to maintenance energy in the deep sea. Alternatively, rhodopsin might be used in a sensory capacity, to allow cells to phototax toward bioluminescent organisms or particulate materials, which have been observed to be bioluminescent [59]. Interestingly, an inward proton pump in a deep-sea bacterial isolate from 800 m in the Southeastern Pacific Ocean was recently reported [60].

Heterologous expression of deep-sea rhodopsins resulted in ion pumping of one deep-water-associated proteorhodopsin originating from SAR11, which contained a unique DTQ-Q motif. Although we did observe a pH change with this unique deep-water rhodopsin, the majority of other unique deep-sea rhodopsin sequences we tested failed to exhibit light-driven proton pumping or retinal binding when expressed in *E. coli*. It is possible that this is due to improper folding or insertion in the *E. coli* membrane or perhaps because *E. coli* growth temperatures or membrane lipid composition are so different from in situ deep-sea conditions. It is also possible that these proteins have inherently lost their retinal binding capability, and have been co-opted for other functions in the cell. Further studies in other organisms and under conditions that more closely approximate the deep-sea environment may provide more insight into the nature and function of these novel deep-sea rhodopsin-related proteins.

If retinal is present at great depths, it may be that at least some of these  proteorhodopsin-like proteins could function as photoreceptors. We found that while retinal biosynthesis proteins were abundant in near surface waters, they were much less prevalent in deeper waters. Specifically, β-carotene and retinal biosynthesis enzymes crtY and blh were nearly undetectable at the greater depths. Interestingly, an alternative retinal biosynthetic route, the diox1 pathway, was detected at depth, which could potentially produce retinal from apo-carotenoids. Also detected at greater depths were genes encoding the enzymes responsible for production of phytoene and lycopene. Even in the absence of known retinal-producing pathways, retinal might be acquired from sinking particles. Measuring retinal and testing the function of these putative diox1 sequences  represents another important avenue to pursue in the future.

Although it is likely some of the rhodopsin sequences found in the deep ocean bind retinal and could act as photoreceptor proteins, the majority may not share this function. The DTT-T motif containing sequences originating from deep-sea dwelling members of the SAR11 clade were the most abundant in the deep-water samples, yet showed no retinal binding or ion pumping function when expressed in *E. coli*. This lack of retinal binding may simply be due to improper folding or membrane insertion. However, changing two motif residues in functional proton pumping proteorhodopsins from surface waters to the DTT-T motif completely abolished their ion pumping activity. This may indicate that these proteins do not act as ion pumps, and it remains unclear whether they act as ion pumps when expressed in their native environment, alternatively act as sensory rhodopsins, or have been adapted to perform some other function in situ.

The majority of both shallow and deep-water proteorhodopsin sequences we identified appeared to originate from different SAR11 types throughout the water column.

Two SAR11 reference rhodopsins were included in the initial phylogenetic analysis (Candidatus Pelagibacter ubique HTCC1062, Candidatus Pelagibacter sp. HTCC7211; Supplemental File 1). These are DTE_L and DTE-Q containing sequences and both fall within the major surface water SAR11 lineage. A number of SAR11 genomes have been published and classified in different subclasses [61–64]. Proteorhodopsin sequences from published SAR11 clade genomes mostly contain green or blue light absorbing rhodopsins all of which map to the surface water proteorhodopsins found in our data set. For example, HTCC1062, HTCC1002, HIMB114, HIMB5, and HIMB59 all encode DTE-L containing proteorhodopsins. HTCC7211 encodes a proteorhodopsin with the DTE_Q motif. Interestingly, one SAR11 genome, which was isolated below the DCM [63], encoded proteorhodopsin genes that map to deep-water SAR11 proteorhodopsins found in our data set. Specifically, alpha proteobacterium SCGC AAA288-N07 encodes a proteorhodopsin with the uncharacterized DTT-T motif that was most abundant in deep waters at Station ALOHA.

The streamlined nature of SAR11 genomes suggests that if these proteorhodopsin genes were nonfunctional, they would be expected to be removed from the genome via mutation and deletional biases. Sequence analysis indicated that these proteorhodopsins do possess a retinal binding pocket, although initial in vitro heterologous expression experiments were unable to confirm this for most DTT-T motif-containing sequences we tested. Future ecological, physiological, and biochemical studies will be required to determine the function of these abundant deep-water proteorhodopsin homologs.

## Electronic supplementary material

Supplemental Legend

Supplemental File 1

Supplemental File 2

Supplemental Table 1

Supplemental Table 2

Supplemental Table 3

Supplemental Figure 1

Supplemental Figure 2

Supplemental Figure 3

Supplemental Figure 4

Supplemental Figure 5
