# Supplementary material for: Proteorhodopsin variability and distribution in the North Pacific Subtropical Gyre
Source: ISME J. 2018 Feb 23;12(4):1047–60. doi: 10.1038/s41396-018-0074-4 (PMC5864233; doi:10.1038/s41396-018-0074-4)
Supplement: Supplementary file 1 — Supplemental Legend [file 41396_2018_74_MOESM1_ESM.docx]

Supplemental File 1. Fasta file of 120 reference rhodopsin sequences.

Supplemental File 2. Fasta file of 1,510 proteorhodopsin sequences used in this study.

Supplemental Table 1. Synthesized proteorhodopsin genes used for functional analysis.

Supplemental Table 2. Synthesized chimeric proteorhodopsin genes used in this study.

Supplemental Table 3. Proteorhodopsin sequence counts from metagenomes with respect to ion pumping and spectral tuning amino acid residues

Supplemental Figure 1. Principal components analysis (PCA) ordination plot of rhodopsin abundance (blue) and environmental data as explanatory variables (red). Other explicative biological variables are in blue. Arrows indicate the direction and magnitude of variables. 83 samples used as observations are colored according to season (dot).

Supplemental Figure 2. *E. coli* cells expressing each rhodopsin gene. These pellets were obtained by centrifuging 200 – 400 ml of the *E. coli* cultured media. Asterisk indicates the samples of which H^+^ pumping activities were observed.

Supplemental Figure 3. Sequence alignment of DTE_Q containing sequences (top 25 sequences) and DTT_T containing sequences (bottom 25 sequences) highlighting the differences in the N-terminus.

Supplemental Figure 4. Functional analyses of synthesized chimeric rhodopsin DNAs. a) Schematic images of chimeric rhodopsin genes. Signal peptide region was predicted by TOPCONS (http://topcons.cbr.su.se/). Numbers in parentheses indicate amino acid no. in PR numbering. b) *E. coli* cells expressing each chimeric DNA by centrifuging 40 ml of the *E. coli* cultured media. c) Light induced pH changes of *E. coli* cell suspensions expressing original rhodopsin or chimeric rhodopsin.

Supplemental Figure 5. Analysis of taxa (a) and ion pumping and spectral tuning motif (b) in the metatranscriptomes.
