# Supplementary figures and images for: Proteorhodopsin variability and distribution in the North Pacific Subtropical Gyre
Source: ISME J. 2018 Feb 23;12(4):1047–60. doi: 10.1038/s41396-018-0074-4 (PMC5864233; doi:10.1038/s41396-018-0074-4)

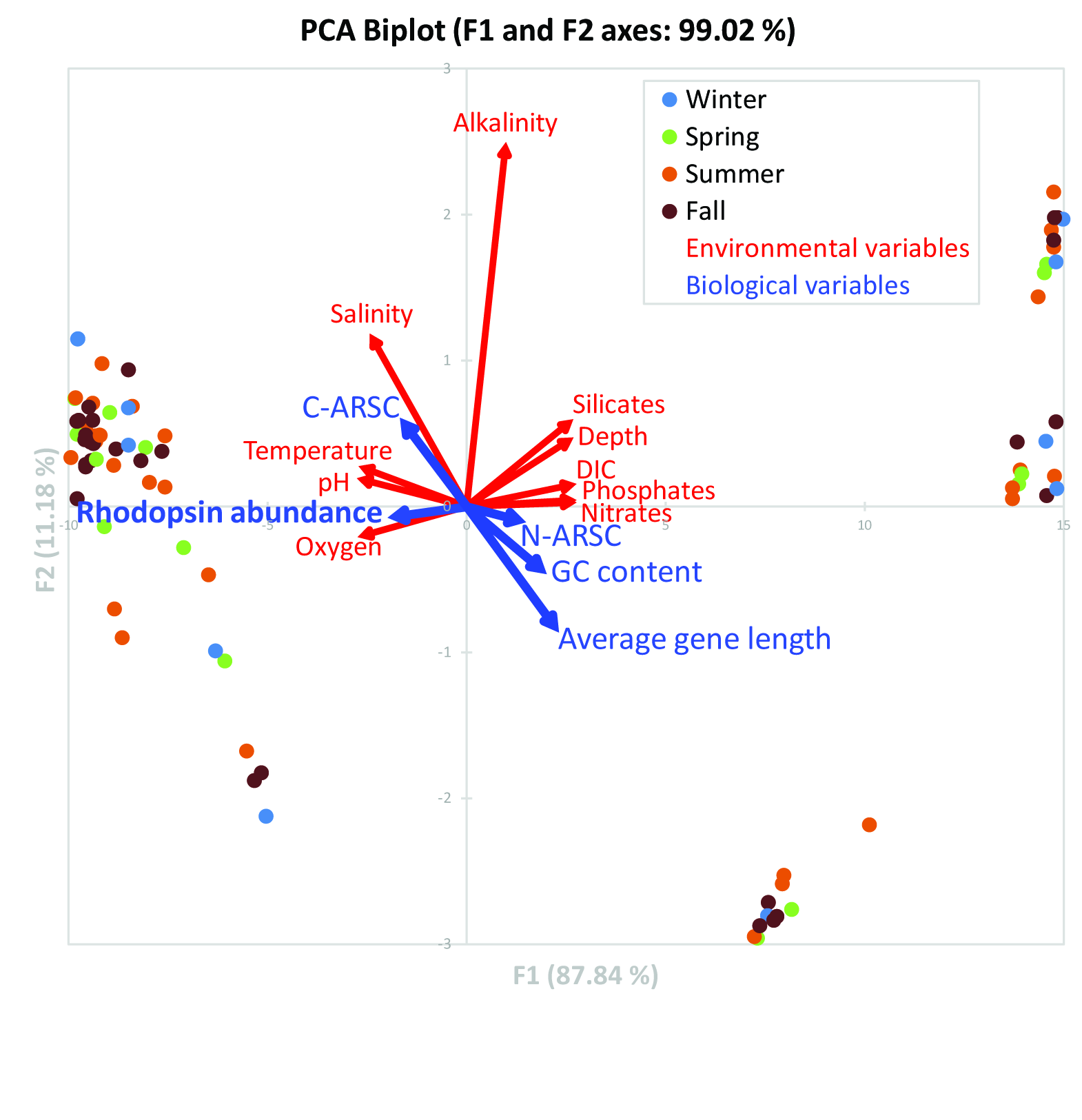

Supplement: Supplementary file 7 — Supplemental Figure 1 [file 41396_2018_74_MOESM7_ESM.tif]

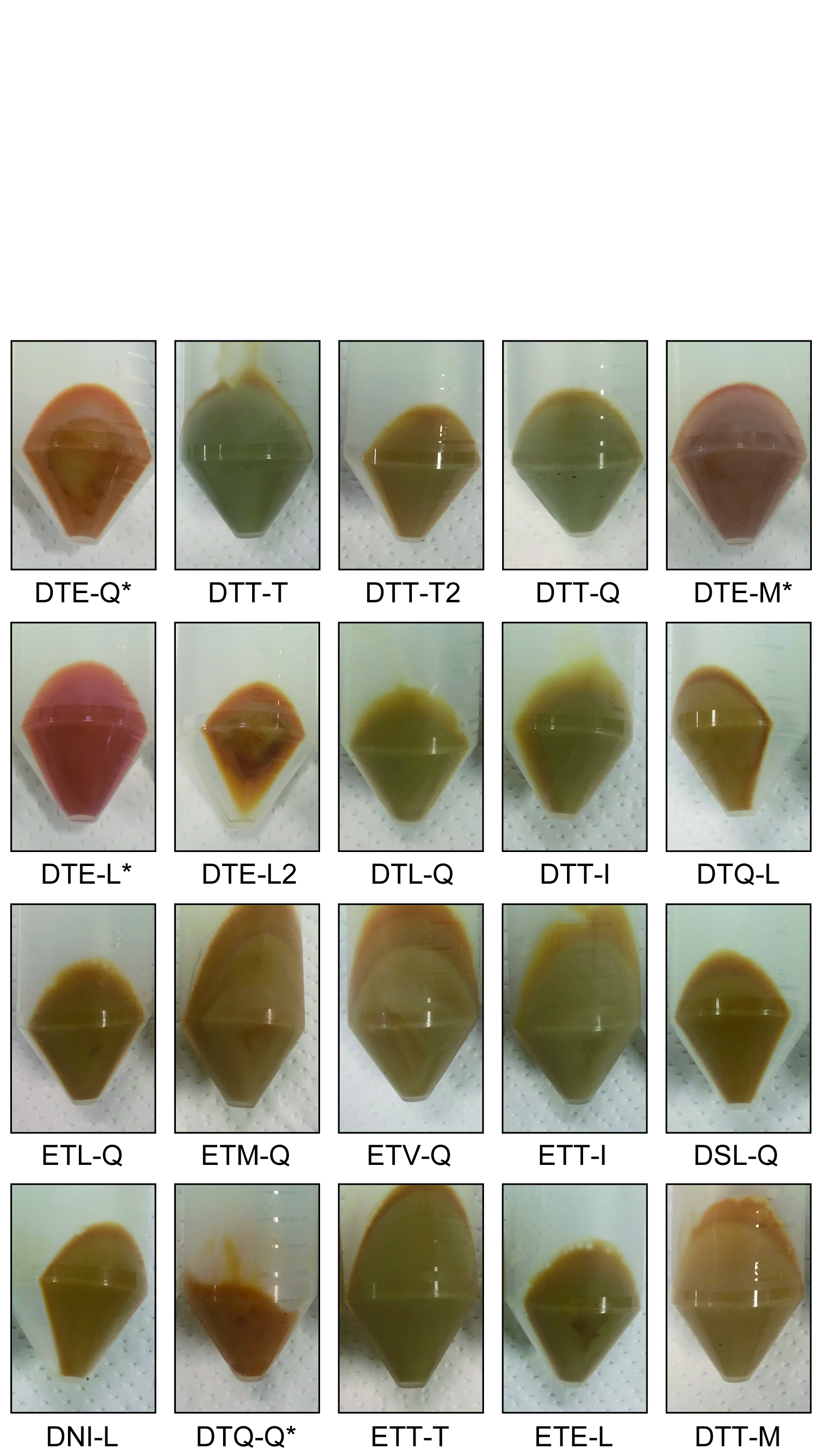

Supplement: Supplementary file 8 — Supplemental Figure 2 [file 41396_2018_74_MOESM8_ESM.tif]

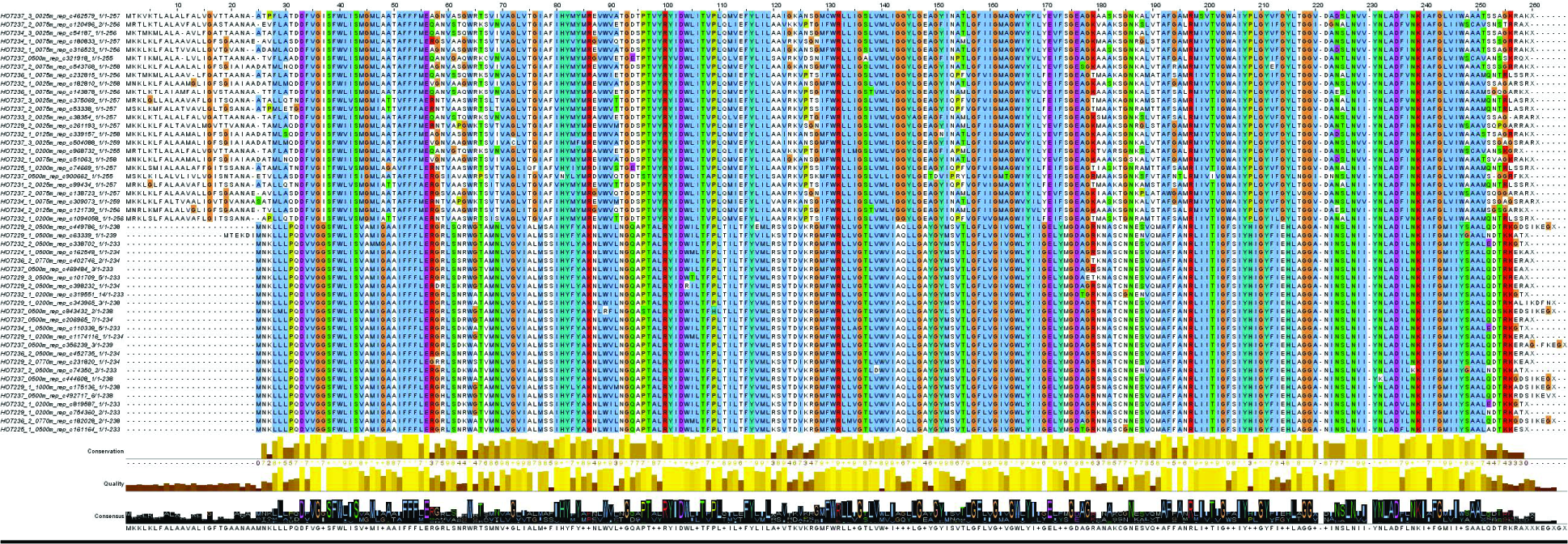

Supplement: Supplementary file 9 — Supplemental Figure 3 [file 41396_2018_74_MOESM9_ESM.tif]

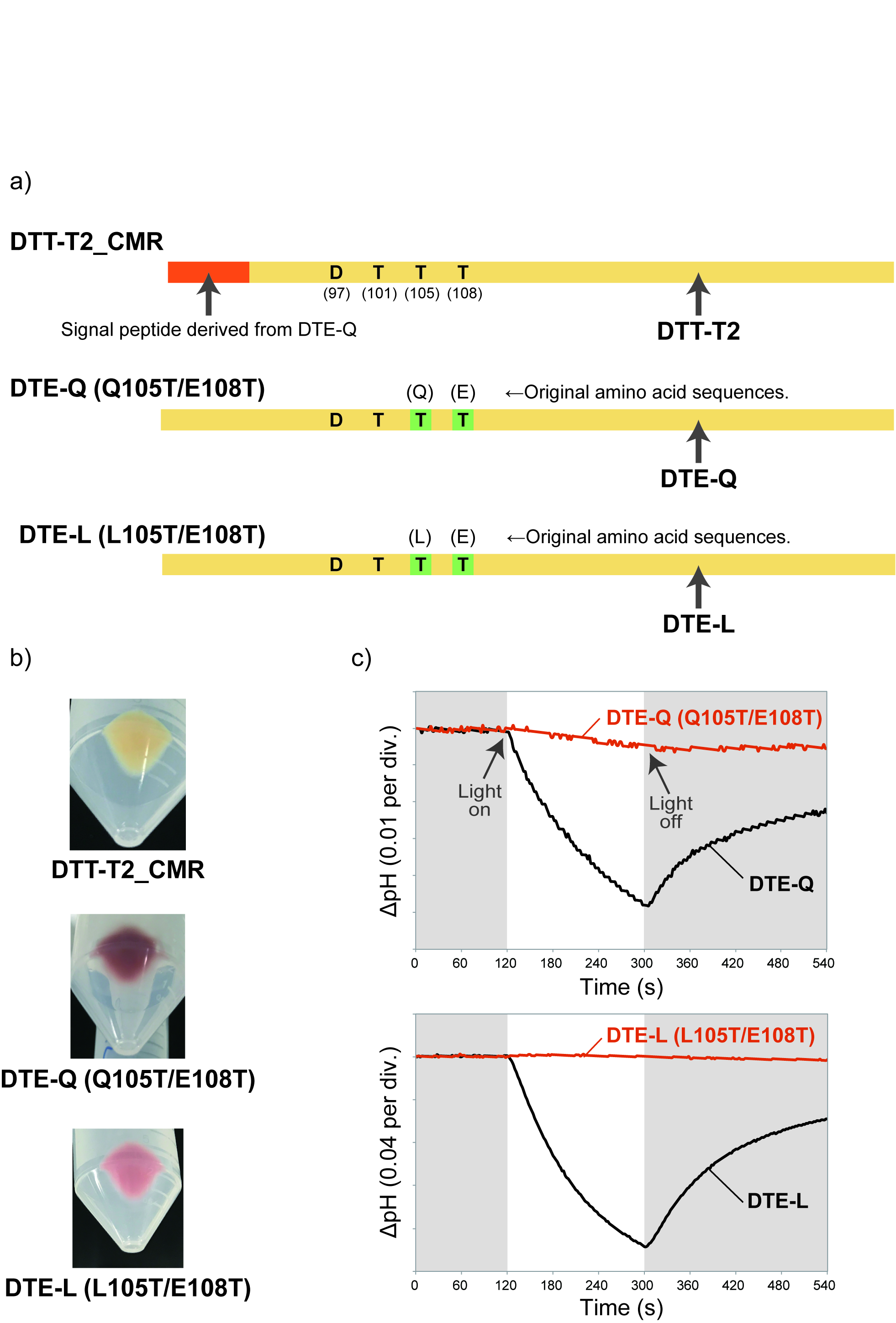

Supplement: Supplementary file 10 — Supplemental Figure 4 [file 41396_2018_74_MOESM10_ESM.tif]

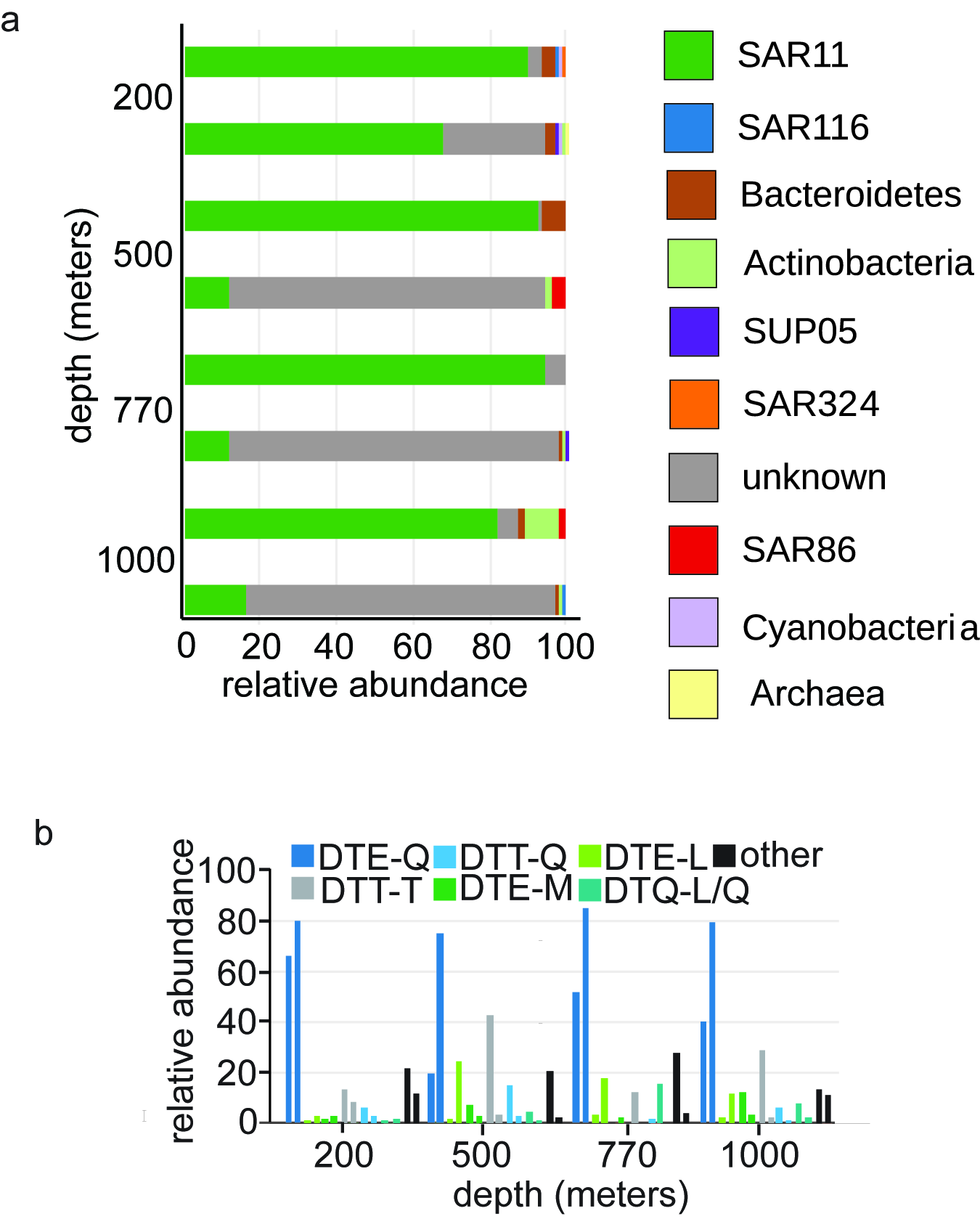

Supplement: Supplementary file 11 — Supplemental Figure 5 [file 41396_2018_74_MOESM11_ESM.tif]
